# Editorial: Molecular Mechanisms Underlying Assembly and Maintenance of the Neuromuscular Junction

**DOI:** 10.3389/fnmol.2021.797832

**Published:** 2021-11-22

**Authors:** Ruth Herbst, Inga Koneczny, Hanns Lochmüller, Laure Strochlic

**Affiliations:** ^1^Center for Pathophysiology, Infectiology and Immunology, Medical University of Vienna, Vienna, Austria; ^2^Division of Neuropathology and Neurochemistry, Department of Neurology, Medical University of Vienna, Vienna, Austria; ^3^Children's Hospital of Eastern Ontario Research Institute, University of Ottawa, Ottawa, ON, Canada; ^4^Division of Neurology, Department of Medicine, The Ottawa Hospital, University of Ottawa, Ottawa, ON, Canada; ^5^Brain and Mind Research Institute, University of Ottawa, Ottawa, ON, Canada; ^6^Sorbonne Université, Institut National de la Santé et de la Recherche Médicale, Institut de Myologie, Centre de Recherche en Myologie, Paris, France

**Keywords:** neuromuscular junction, skeletal muscle, motor neuron, neuromuscular disease, experimental disease models, muscle weakness, aging

The Frontiers Research Topic “*Molecular mechanisms underlying assembly and maintenance of the neuromuscular junction*” highlights recent progress in the field of neuromuscular development and maintenance in health and disease. Every voluntary movement such as breathing, walking and eating relies on the appropriate function of the neuromuscular junction (NMJ), therefore much of our behavior, well-being, and productivity are governed by this specialized synapse. NMJ dysfunction occurs in many different diseases affecting the neuromuscular system, including motor neuron diseases, muscular dystrophies, autoimmune diseases, and congenital myasthenic syndromes (Li et al., 2018). Most of these disorders are difficult to treat and can be life threatening. Moreover, NMJ maintenance plays a key role in weakened muscle function during aging (Hunter et al., 2016). It is therefore of central importance to understand the mechanisms of NMJ development, maintenance, and function under healthy and pathological conditions to develop new therapeutic strategies for patients and an aging society. In this special issue, we combined comprehensive reviews of new emerging concepts together with original articles investigating the complex molecular mechanisms that govern the formation, maintenance, and integrity of the NMJ to its dysfunction in aging and diseases.

The NMJ is a complex structure, formed by a highly organized interplay between motoneuron, muscle endplate, and perisynaptic Schwann cells (Sanes and Lichtman, 2001; Li et al., 2018). Its development and functional maturation as well as physiological adaptation throughout life require a coordinated sequence of molecular mechanisms. Cruz et al. provide a comprehensive review about the complexity of NMJ development covering mechanisms involved in synapse formation and maintenance as well as in disease and aging. The muscle-type nicotinic acetylcholine receptor (AChR) is the key component at the post-synaptic muscle membrane that is activated upon presynaptic neurotransmitter release and controls muscle response. High concentration and localized AChR expression are prerequisites for efficient neurotransmission. Subunit composition changes during development and influences properties of the AChR. Cetin et al. summarize the structural and functional differences between the fetal and adult AChR isoforms and their distinct roles during NMJ formation and function. The study by Rudell et al. characterized the MX-helix in the β and δ subunits of the AChR to show their role in receptor assembly and surface expression, thereby explaining the functional consequences of mutations found in congenital myasthenic syndromes. Synaptic nuclei closely associated with the post-synaptic membrane express specific genes and contribute to the development and function of the NMJ. Huang et al. used the RiboTag techniques to perform a large-scale gene expression profiling of transcripts present at NMJs in fast-twitch muscle. The authors found an enrichment of known and novel genes that are part of different networks such as diacylglycerol and phosphatidylinositol signaling or extracellular matrix components, thus expanding the group of molecular players involved in NMJ development.

NMJ maturation and maintenance are essential for precise and refined muscle contraction. The size of the NMJ increases, its morphology and molecular composition changes and high AChR density has to be maintained. The molecular mechanisms that regulate maturation and maintenance remain largely unresolved. Bernadzki et al. identified the guanine nucleotide factor Arhgef5 as binding partner of α-Dystrobrevin, which is part of the dystrophin-associated glycoprotein complex and important for NMJ maintenance. Mice lacking Arhgef5 expression in the muscle show a loss of NMJ structural integrity and a reduction of active RhoA and Cdc42 pointing to the importance of actin dynamics for NMJ maintenance. Eiber et al. found in a knock-out mouse model for the intermediate filament protein desmin that NMJs are structurally and functionally impaired. These findings support previous reports of patients with myasthenic syndrome in addition to the classical occurrence of myopathy and cardiomyopathy caused by mutation in desmin. NMJ perturbations have been critically linked to age-related loss of muscle mass and function. The mechanisms responsible for NMJ destabilization during aging remain elusive but the TOR (Target of Rapamycin) pathway has drawn attention due to its crucial role in proteostasis. Castets et al. discuss the recent insights on TOR and its role in NMJ stability and function. Badawi and Nishimune focus on age-dependent functional and morphological decline of human NMJs. Aging processes in mouse models of accelerated aging are compared to human phenotypes and potential intervention approaches to attenuate age-dependent NMJ dysfunction and sarcopenia are discussed.

Neuromuscular disorders are a large group of heterogeneous disorders that can affect the function of skeletal muscles, peripheral nerves or different components of the NMJ, resulting in skeletal muscle weakness, myoclonus, muscle stiffness, or myalgia. They are a diverse group of diseases with greatly varying parameters such as age of onset, affected muscle groups, pathogenic mechanisms, disease severity, co-morbidities, and treatment options. Unraveling the underlying molecular mechanisms of NMJ development and function is key to our understanding of pathogenic mechanisms and essential for the development of novel diagnostic/prognostic assays and therapies.

Three original reports highlight the importance of genetic defects in neuromuscular transmission disorders, so-called congenital myasthenic syndromes (CMS). Della Marina et al. report on 32 patients with defects in 8 different CMS-genes, who were followed for a median time of 12.8 years at a single clinical center in Germany. Karmouch et al. provide new insights into the signaling pathways that are regulated by ColQ-deficiency and highlight a role for HuR and p38 in mRNA stability in a mouse model of CMS. Finally, Spendiff et al. treated CMS model mice with a genetic defect in the *AGRN* gene using a small agrin compound, which restored post-synaptic structure and improved muscle strength through normalization of muscle fiber composition and the prevention of atrophy. Pathogenic autoantibodies against neuromuscular proteins interfere with NMJ structure and function in myasthenia gravis (MG). Takamori reviews the known intra- and extracellular autoantibodies in MG and how they affect AChR clustering, trans-synaptic homeostasis and synaptic stability, while Cao et al. provide an in-depth state-of the art of the clinical features, pathophysiology, and treatment of MG with autoantibodies against MuSK. A subgroup of MG with autoantibodies to the AChR is also strongly associated with the presence of thymoma (TAMG), which may play a role in the immunopathogenesis, as the thymus is essential to uphold central tolerance mechanisms and remove autoreactive T-cells that could initiate an immune reaction against NMJ proteins (Marx et al., 2013). Recent studies suggest that thymomas in TAMG may be of a distinct molecular subtype of thymic epithelial tumors, but little is known about the epigenetic modifications of thymomas in TAMG. Coppedè et al. investigated blood and thymoma tissues from 69 TAMG patients and studied the methylation pattern of tumor suppressor and DNA repair genes but did not observe any differences in the methylation between thymoma tissue and unaffected thymus tissue or any correlation between clinic, methylation and histopathology.

Treatment of neuromuscular disorders remains challenging and the development of stem cell-based therapies represents a promising alternative to current treatment options. Rigon et al. tested adipose-derived stromal cells for their potential to support NMJ and muscle regeneration. The authors find a short-term beneficial effect on NMJ regeneration but also observed late phase muscle necrosis and fibrosis induced by an inflammatory response. Myasthenic weakness may be exacerbated by a range of drugs that can interfere with NMJ function, pre- or post-synaptically, including antibiotics, cardiovascular drugs, volatile anesthetics, corticosteroids, lithium, magnesium and other drugs, and a range of drugs and treatments are under suspicion to precipitate *de novo* myasthenic syndromes, such as chloroquine, D-penicillamine, or immune checkpoint inhibitors. Krenn et al. provide an overview over drugs and treatments that influence or possibly induce myasthenic weakness, thereby increasing awareness of adverse treatment effects.

Within this special issue, articles discuss NMJ physiology and highlight some of the complex coordinated events critical for synaptic formation and homeostasis as well as the pathogenic mechanisms underlying NMJ dysfunction. We therefore believe that this special issue provides an excellent overview on current knowledge and future challenges in the field of the neuromuscular system.

## Dedicated to Wes Thompson

The research community lost Wesley J. Thompson, one of the pioneers of neuromuscular research in 2019. He contributed seminal work on the role of nerve activity and Schwann cells during NMJ development discussed in detail by Lee and Rimer. Wes was a passionate scientist, a great mentor and wonderful person. He is deeply missed and we would like to honor his loss by dedicating this special issue to our dear friend and colleague Wes Thompson.

## Author Contributions

All authors listed have made a substantial, direct, and intellectual contribution to the work and approved it for publication.

## Funding

RH was supported by the Austrian Science Fund (FWF), projects P28485-B24 and P31199-B27. IK was funded by a Hertha Firnberg project grant by the Austrian Science Fund (FWF), project number T996-B30. HL receives support from the Canadian Institutes of Health Research (Foundation Grant FDN-167281), the Canadian Institutes of Health Research and Muscular Dystrophy Canada (Network Catalyst Grant for NMD4C), the Canada Foundation for Innovation (CFI-JELF 38412), and the Canada Research Chairs program (Canada Research Chair in Neuromuscular Genomics and Health, 950-232279). LS was supported by grants from AFM-Téléthon (grant ID 22691) and the Institut National de la Santé et de la Recherche Médicale (INSERM).

## Conflict of Interest

The authors declare that the research was conducted in the absence of any commercial or financial relationships that could be construed as a potential conflict of interest.

## Publisher's Note

All claims expressed in this article are solely those of the authors and do not necessarily represent those of their affiliated organizations, or those of the publisher, the editors and the reviewers. Any product that may be evaluated in this article, or claim that may be made by its manufacturer, is not guaranteed or endorsed by the publisher.
